# Timing of Closure of a Protective Loop-Ileostomy Can Be Crucial for Restoration of a Functional Digestion

**DOI:** 10.3389/fsurg.2022.821509

**Published:** 2022-03-28

**Authors:** Jens M. Werner, Paul Kupke, Matthias Ertl, Sabine Opitz, Hans J. Schlitt, Matthias Hornung

**Affiliations:** Department of Surgery, University Hospital Regensburg, Regensburg, Germany

**Keywords:** protective loop-ileostomy, enterostomy, closure surgery, surgical complications, dysfunctional digestion

## Abstract

**Introduction:**

Protective loop-ileostomy is one of the most common interventions in abdominal surgery to provide an alternative intestinal outlet until sufficient healing of a distal anastomosis has occurred. However, closure of a loop-ileostomy is also associated with complications. Thus, knowledge of the optimal time interval between primary and secondary surgery is crucial.

**Methods:**

Data from 409 patients were retrospectively analyzed regarding complications and risk factors in closure-associated morbidity and mortality. A modified Clavien-Dindo classification of surgical complications was used to evaluate the severity of complications.

**Results:**

A total of 96 (23.5%) patients suffered from postoperative complications after the closure of the loop-ileostomy. Early closure within 150 days from enterostomy (*n* = 229) was associated with less complications (*p* < 0.001^**^). Looking at the severity of complications, there were significantly more (*p* = 0.014^*^) mild postoperative complications in the late closure group (>150 days). Dysfunctional digestive problems—either (sub-) ileus (*p* = 0.004^*^), diarrhea or stool incontinence (*p* = 0.003^*^)—were the most frequent complications associated with late closure. Finally, we could validate in a multivariate analysis that “time to closure” (*p* = 0.002^*^) is independently associated with the development of complications after closure of a protective loop-ileostomy.

**Conclusion:**

Late closure (>150 days) of a loop-ileostomy is an independent risk factor in post-closure complications in a multivariate analysis. Nevertheless, circumstances of disease and therapy need to be considered when scheduling the closure procedure.

## Introduction

Installation of an artificial bowel output (enterostomy) to circumvent an intestinal obstruction can be traced back to ancient times with first records of a surgical ileostomy, ranging back to 1879 (1). Today, many oncologic and non-oncologic diseases involving intra-abdominal organs demand the installation of an enterostomy during disease owed to complications, such as perforation, obstruction, compression, or infection of the intestine (1–3). Protective (loop-) ileostomy is one of the most common interventions in abdominal surgery to provide—in conjunction with the attached stoma appliance—an alternative intestinal outlet (2).

Procedures of surgical enterostomy are principally reversible, and, especially, a protective loop-ileostomy is generally intended to be only temporary until sufficient healing of a distal anastomosis has occurred (2). However, not only the feasibility of stoma closure but also the timing is a relevant question that is a decisive factor influencing patient-related physical and psychological outcomes (4, 5). Until recently, abdominal surgeons have widely agreed upon a temporizing strategy when confronted with a decision toward or against early closure of a protective loop-ileostomy (6–8). However, current literature suggests that a belated closure of a protective loop-ileostomy—even though lacking a consistent and consensual critical cut-off—might be associated with higher morbidity and mortality, thus, suggesting some prognostic risk factors in post-closure complications (2–5, 9–12). In our hospital, we aim to perform the stoma reversal procedure within 3–6 months.

Because of the inconsistent and yet sparsely conducted research, this study wants to validate those recent observations and aims at confining the optimal time interval to ameliorate adverse outcomes after the closure of protective loop-ileostomy.

## Methods

In a retrospective analysis, 409 patients with the closure of a protective loop-ileostomy—as the only inclusion criterion—at the University of Regensburg medical center were included. The time of primary surgery covered a period from January 2000 to August 2012. Patient demographics, primary diagnosis, and indication for enterostomy as well as details of the circumstances of enterostomy creation and the primary surgery, and stoma-related complications during and after a hospital stay, as well as information about ileostomy closure and follow-up care, were recorded by means of a hospital-internal questionnaire.

The widely approved Clavien-Dindo classification of surgical complications was applied for the ranking of adverse perioperative outcomes (13, 14). Grade I represents mild complications and comprises any deviation from the normal postoperative course without the need for pharmacological treatment or surgical, endoscopic, or radiological interventions. The present modification condensed Clavien-Dindo Grades II and III to the new category moderate complications that summarize those unwanted events that indicate any further intervention. Severe complications (Clavien-Dindo Grade IV) contain life-threatening conditions requiring ICU management and/or re-operation. Lethal outcomes correspond to Clavien-Dindo Grade V. Complication rates were operationalized as a proportion of patients with at least one adverse sequelae of the respective population.

Statistical analysis was conducted with IBM SPSS Statistics 20. Data were checked for normal distribution with the Kolmogorov-Smirnov-Test. Fisher's Exact test or Chi-Square test served for comparison of nominal values; risk factors were analyzed with univariate and multivariate logistic regression. When reaching a two-sided α-error level of *p* < 0.05, statistical significance was assumed.

This clinical research project was assessed and approved by the local Ethical Committee of the University of Regensburg medical center under reference No. 18-104-899.

## Results

### Late Closure of a Protective Loop-Ileostomy Has a Higher Risk of Complications

**Figure 1:**
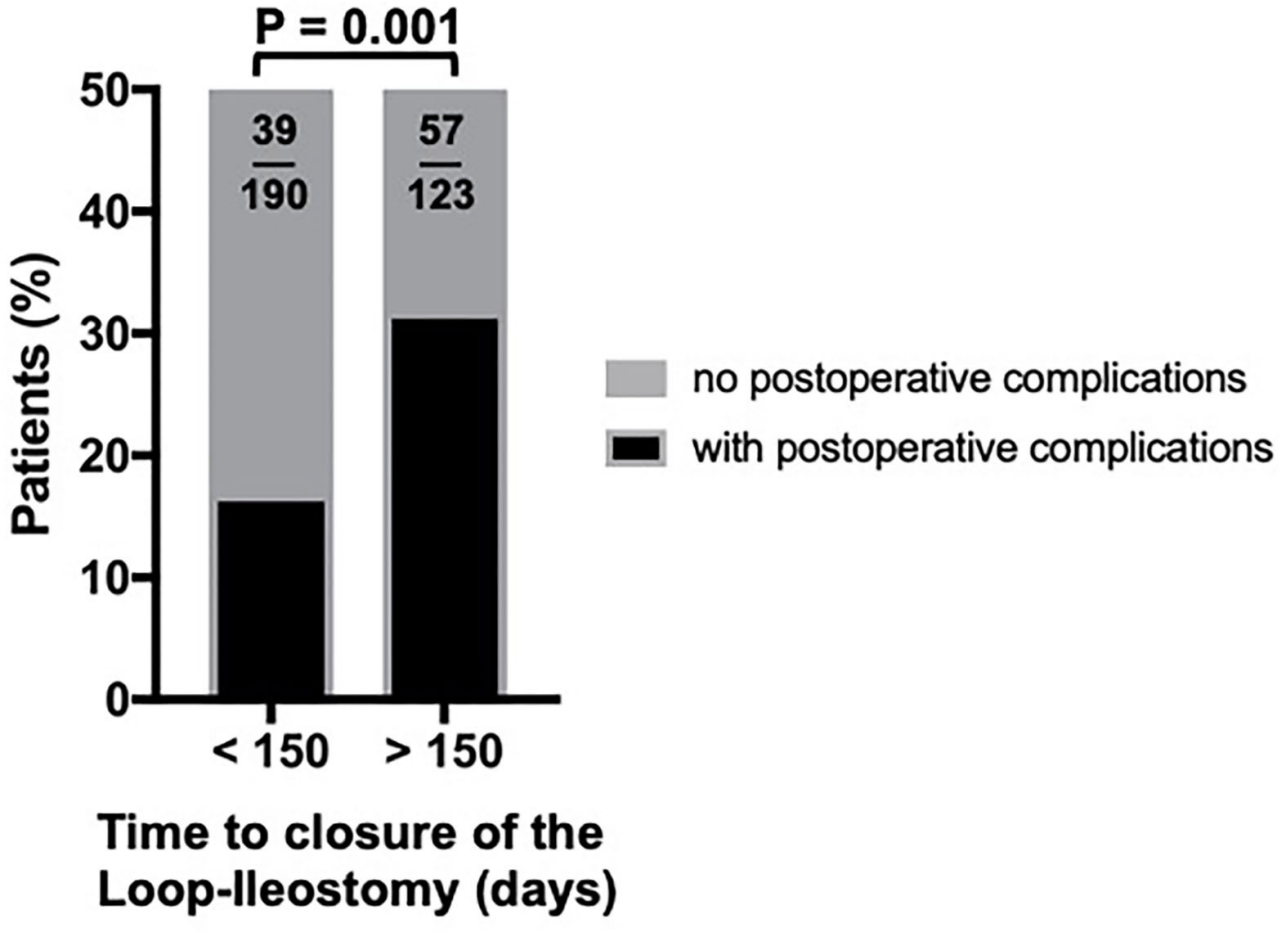
Complications depending on the time to closure of the protective loop-ileostomy.

**Figure 2:**
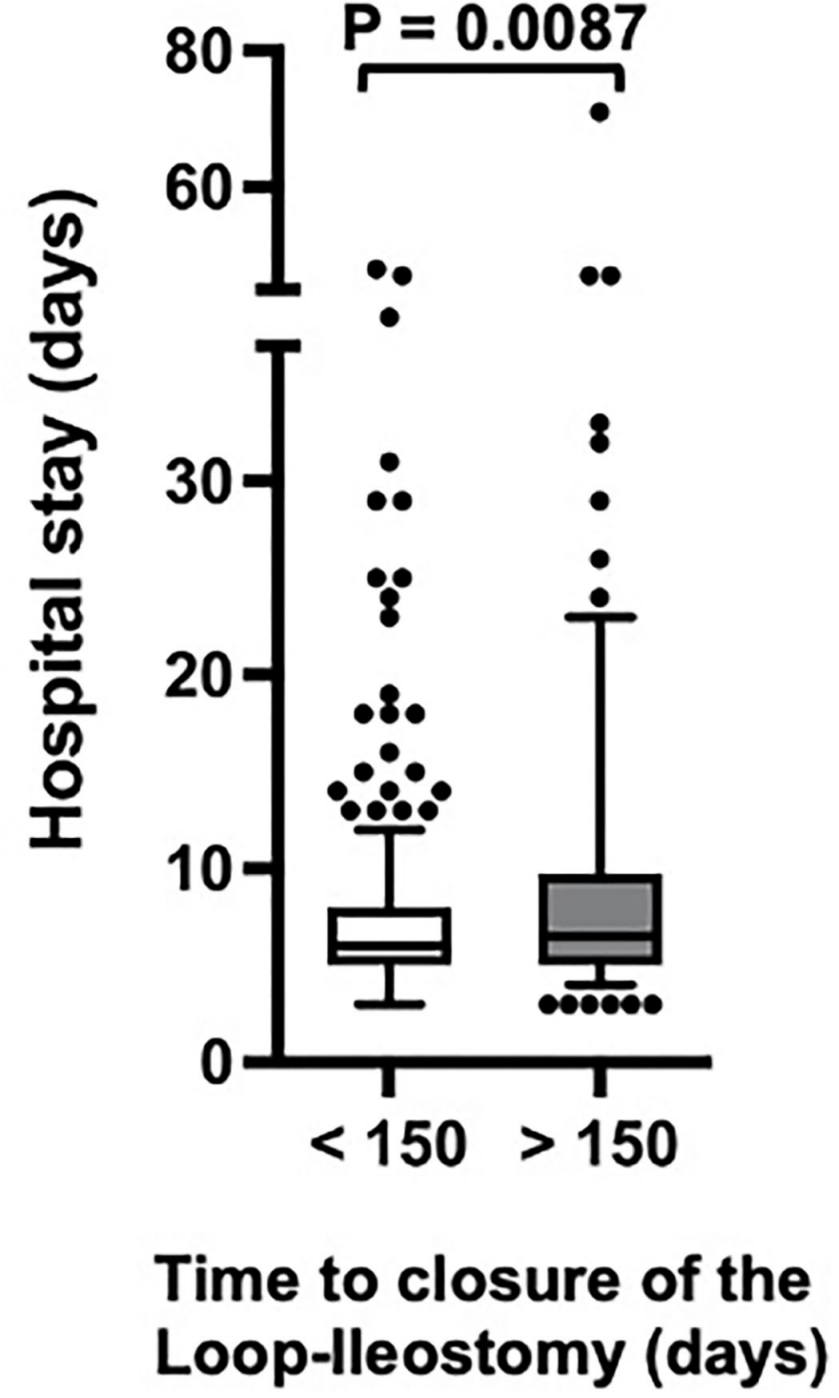
Hospital stay depending on the time of closure of the protective loop-ileostomy. Statistic: non-parametric Mann–Whitney test.

In our study population, closure of a protective loop-ileostomy was feasible in 86.8% (data not shown). That means, at the same time, 13.2% of all cases failed to be reversed, and a temporarily intended enterostomy might have become a permanent one (death, *n* = 39; lost to follow-up, *n*=16; refused any further surgical intervention, *n* = 4). For the 409 patients included in our further analyses, the average time from the primary procedure to the closure of the protective loop ileostomy was 1,674 days (median = 136 days). A negative and unwanted post-closure outcome with postoperative complications affected nearly every fourth closure procedure (23.5%). We found that waiting more than 90 days (*p* = 0.032^*^) or 120 days (*p* = 0.012^*^) was already associated with a significantly higher rate of postoperative complications (data not shown). However, as shown in Figure 1 and Table 1, especially scheduling the closure procedure after 150 days from the initial procedure, made a decisive difference concerning negative outcomes after loop-ileostomy closure (31.7 vs. 17.%; *p* = 0.001^**^) compared to an early closure within 150 days. Furthermore, as shown in Figure 2, patients with a late closure procedure also had a significantly longer hospital stay (median, 6 vs. 6.5 days and IQR, 3 vs. 4.75 days; *p* = 0.0087).

**Table 1:** Complications after protective loop-ileostomy closure.

	**Total [*n* = 409]**	**Closure <150 days [*n* = 229]**	**Closure > 150 days [*n* = 180]**	***p*-value**
Complications [% (*n*)]	23.5 (96)	17.0 (39)	31.7 (57)	<0.001**
**Quality of complications [% (** ***n*** **)]**				
Anastomosis insufficiency	3.7 (15)	2.2 (5)	5.6 (10)	0.072 (ns)
(Sub-) Ileus	8.3 (34)	4.8 (11)	12.8 (23)	0.004*
Diarrheaorstoolincontinence	9.5 (39)	5.7 (13)	14.4 (26)	0.003*
Fistula orabscess	2.9 (12)	1.7 (4)	4.4 (8)	0.109 (ns)
Injury of other intraabdominal organs	0.7 (3)	1.3 (3)	–	0.123 (ns)
Impaired wound healing	4.2 (17)	3.9 (9)	4.4 (8)	0.796 (ns)
Hernia	0.7 (3)	0.9 (2)	0.6 (1)	0.708 (ns)
**The severity of complications [% (** ***n*** **)]**				
I° (mild)	11.2 (46)	7.9 (18)	15.6 (28)	0.014*
II° (moderate)	6.6 (27)	4.8 (11)	8.9 (16)	0.099 (ns)
III° (severe)	5.6 (23)	4.4 (10)	7.2 (13)	0.213 (ns)

*p <0.05, **p <0.001.

### Reasons for Late Closure of a Protective Loop-Ileostomy

Having established that timing of the closure of a protective loop-ileostomy had a significant impact on the rate of postoperative complications, we further analyzed potential reasons for a belated closure procedure to find any distribution bias between the early (<150 days) and late (>150 days) closure group. As shown in Table 2, the patients who received an early closure (mean = 57.5 years, SD = 14.6 years) were—on average—significantly younger than the patients whose enterostomy was closed after 150 days (mean = 60.3 years, SD = 11. years; *p* = 0.028^*^). However, the ratio between young and old patients with enterostomy below or above 60 years of age was equally distributed between both groups (*p* = 0.122). Furthermore, there was no significant difference in the gender distribution between the early (men: 71.2%; women: 28.8%) and late (men: 73.3%; women: 26.7%) closure groups (*p* = 0.630).

**Table 2:** Distribution of risk factors in the overall population and within the subgroups of early and late closure.

	**Total [*n* = 409]**	**Closure <150 days [*n* = 229]**	**Closure > 150 days [*n* = 180]**	***p*-value**
Age [M (SD)]	59.5 years (13.1 years)	57.5 years (14.6 years)	60.3 years (11.0 years)	0.028*
**Age groups [% (** ***n*** **)]**				0.122 (ns)
<60 years	45.97 (188)	49.34 (113)	41.67 (75)	
>60 years	54.03 (221)	50.66 (116)	58.33 (105)	
**Sex [% (** ***n*** **)]**				0.630 (ns)
Men	72.13 (295)	71.18 (163)	73.33 (132)	
Women	27.87 (114)	28.82 (66)	26.67 (48)	
**Primary diagnosis[% (** ***n*** **)]**				0.001**
Rectal cancer	73.35 (300)	65.50 (150)	83.33 (150)	<0.001**
Ulcerative colitis	13.20 (54)	17.90 (41)	7.22 (13)	0.002*
Peritoneal carcinomatosis	7.58 (31)	10.04 (23)	4.44 (8)	0.034*
Sigmoid (colon) cancer	5.87 (24)	6.55 (15)	5.00 (9)	0.508 (ns)
**Primary surgery[% (** ***n*** **)]**				0.491 (ns)
Colectomy, hemicolectomy, sigma resection	7.34 (30)	7.42 (17)	7.22 (13)	0.938 (ns)
Extraperitoneal rectum resection (cytoreductive surgery)	3.42 (14)	4.37 (10)	2.22 (4)	0.236 (ns)
Low anterior rectum resection, proctocolectomy	89.24 (365)	88.21 (202)	90.56 (163)	0.447 (ns)
**Reconstruction[% (** ***n*** **)]**				0.277 (ns)
End to end	84.60 (346)	88.65 (203)	79.44 (143)	
Side to side	11.25 (48)	10.04 (24)	12.78 (24)	
**Suture technique[% (** ***n*** **)]**				0.359 (ns)
Running suture	91.93 (376)	94.76 (217)	88.33 (159)	
Single stitches	2.44 (10)	3.06 (7)	1.67 (3)	
Stapler	0.49 (2)	0.87 (2)	0 (0)	
Adjuvant chemotherapy [% (*n*)]	49.88 (204)	28.33 (97)	59.44 (107)	0.014*

Missing documentation about surgical reconstruction; *p < 0.05, **p < 0.001.

Regarding the primary diagnosis, the patients with rectal cancer significantly more often (83.3%) underwent a late closure after 150 days from primary surgery compared to the patients with other diagnoses (*p* < 0.001^**^), while the closure of protective loop-ileostomy in the patients with ulcerative colitis (17.9%; *p* = 0.002^*^) or peritoneal carcinomatosis (10%; *p* = 0.034^*^) was significantly more frequently conducted early within 150 days after enterostomy. Only the patients with (sigmoid) colon cancer were equally distributed between the groups of early and late closure (*p* = 0.508).

The corresponding primary surgical interventions, however, did not significantly differ in the distribution between early or late closure (*p* = 0.491)—neither for (hemi-) colectomy and sigma resection (*p* = 0.938), nor for extraperitoneal rectum resection (*p* = 0.236) or low anterior rectum resection and proctocolectomy (*p* = 0.447). Furthermore, neither the reconstruction technique (end to end or side to side), nor the suture technique was significantly different between the two groups. However, the patients in the late closure group frequently received significantly adjuvant chemotherapy before the closure procedure was performed (*p* = 0.0141).

### Late Closure of a Loop-Ileostomy Is Associated With Digestive Dysfunction

Next, we analyzed the severity of post-closure complications based on a slightly modified Clavien-Dindo classification. As shown in Table 1, digestive dysfunctions occurred significantly more often in the patients with a late closure (>150 days): (sub-) ileus (4.8 vs. 12.8%; *p* = 0.004^*^) or diarrhea and stool incontinence (5.7 vs. 14.4%; *p* = 0.003^*^) affected the patients with a belated closure more often. Other unwanted outcomes, such as formation of fistulas or abscesses (1.7 vs. 4.4%; *p* = 0.109), injury of other intra-abdominal organs (1.3 vs.0.0%; *p* = 0.123), insufficient wound healing (3.9 vs. 4.4%; *p* = 0.796) or development of an abdominal wall hernia (0.9 vs.0.6%; *p* = 0.708), were found with a similar contribution between both groups. Moreover*, the* severity of the post-closure complications was associated with the timing of the closure procedure. Early stoma closure within 150 days from primary surgery was associated with significantly less mild (7.9 vs. 15.6%; *p* = 0.014^*^) complications. The categories of moderate (4.8 vs. 8.9%; *p* = 0.099) or severe complications (4.4 vs. 7.2%; *p* = 0.213), however, resembled similar distribution between both groups without lethal complications.

### Risk Factors in Post-closure Complications

In a final step, we wanted to link certain associated factors with post-closure complications. We found “time to clo” (*p* < 0.001^**^) and “sex” (*p* = 0.045^*^) as significant risk factors in the development of post-closure complications in a univariate logistic regression analysis as demonstrated in Table 3. Late closure of the protective loop-ileostomy after 150 days was associated with up to 12 times elevated risk for complications compared to early closure within 150 days from primary surgery (OR: 0.443; CI, 95%: 0.078–0.706) and the risk for men tripled that of women (OR: 0.566; CI, 95%: 0.324–0.988). Finally, we could substantiate that the timing of the closure is still a significant risk factor (*p* = 0.002^*^), even when controlled for gender, in a multivariate logistic regression analysis as shown in Table 4.

**Table 3:** Univariate analysis of risk factors in complications after closure of the protective loop-ileostomy.

	**OR**	**CI 95%**	***p*-value**
**Time to closure**			0.001**
<150 days	0.443	0.078-0.706	
>150 days	1		
**Age groups**			0.231 (ns)
<60 years	0.753	0.474–1.197	
>60 years	1		
**Sex**			0.045*
Men	1		
Women	0.566	0.324–0.988	
**Primary diagnosis**			0.131 (ns)
Rectal cancer	1		
Ulcerative colitis	0.636	0.305–1.323	0.226 (ns)
Peritoneal carcinomatosis	0.300	0.089–1.013	0.053 (ns)
Sigmoid (colon) cancer	0.559	0.186–1.687	0.302 (ns)
**Primary surgery**			0.645 (ns)
Colectomy, hemicolectomy, sigma resection	0.630	0.234–1.694	0.359 (ns)
Extraperitoneal rectum resection (cytoreductive surgery)	858	0.234–3.147	0.818 (ns)
Low anterior rectum resection, proctocolectomy	1		

**Table 4:** Multivariate analysis of risk factors in complications after closure of the protective loop-ileostomy.

	**OR**	**CI 95%**	***p*-value**
**Time to closure**			0.002*
<150 days	0.468	0.289–0.757	
>150 days	1		
**Age groups**			0.562 (ns)
<60 years	0.859	0.514–1.436	
>60 years	1		
**Sex**			0.121 (ns)
Men	1		
Women	0.632	0.353–1.129	
**Primary diagnosis**			0.981 (ns)
Rectal cancer	1		
Ulcerative colitis	0.932	0.397–2.187	0.872 (ns)
Peritoneal carcinomatosis	0.000	0.000	0.998 (ns)
Sigmoid (colon) cancer	0.712	0.146–3.480	0.675 (ns)
**Primary surgery**			0.962 (ns)
Colectomy, hemicolectomy, sigma resection	0.814	0.191–3.472	0.780 (ns)
Extraperitoneal rectum resection (cytoreductive surgery)	0.000	0.000	0.998 (ns)
Low anterior rectum resection, Proctocolectomy	1		

## Discussion

The best timing of the closure of a protective loop-ileostomy is yet a quite inconclusive issue with many considerations being insufficiently addressed. Finding the “sweet spot” is further aggravated, because either a hasty or a delayed closure is accompanied by a tremendous risk of post-closure complications (5, 6, 15), amounting to 23.5% in total in the present study. Here, the number of adverse outcomes after protective loop-ileostomy closure was strongly associated with the time interval between primary surgery and closure of the enterostomy. When bowel continuity was restored within 150 days, complications occurred in 17%. Waiting more than 150 days for the closure procedure was associated with complications in almost every third case (31.7%).

The time between installation and closure of a protective loop-ileostomy is often substantially longer than initially planned. Completion of adjuvant chemotherapy is usually the leading argument against the closure of an enterostomy (7, 8). But waiting too long might result in medical, surgical, and psychological impairments (2, 3): Electrolyte derangements, dehydration, and malnutrition such as parastomal skin irritations can be found frequently in patients with a protective loop-ileostomy, and problems such as parastomal herniation, obstruction, or ileus may require surgical intervention (1, 16). Besides, an artificial bowel output disturbs activities of daily living, often leading to a diminution of health-related quality of life, and it changes the self-concept, which, in turn, could lower the patient's self-esteem (17, 18).

As we could demonstrate, dysfunctional complications such as either (sub-) ileus or diarrhea and stool incontinence might not only occur due to a prolonged loop-ileostomy but also as a result of a belated closure. Even though the severity of post-closure complications was relatively low and did not differ between groups in the categories of moderate and severe negative outcomes, mild complications were found significantly more frequent in patients with a late closure of a protective loop-ileostomy. Our data are in line with reports from Abdalla and Scarpinata (19) as well as Hughes et al. (20), who accounted for the negative impact of a delayed closure more than 6 months after index surgery on the rate of post-closure complications in a small cohort, whereas Zhen et al. (21) could not substantiate the inferiority of a late closure operation. The authors observed a comparable number of adverse outcomes for patients with a closure beyond 6 months from primary surgery, but this study group actually received more adjuvant chemotherapy cycles and might, thus, even have a better prognosis than patients with an early closure. Li and Ozuner (22) investigated a time interval of more or <3 months between enterostomy and stoma closure. Findings revealed no relevant intergroup differences.

Closure of a protective loop-ileostomy has to be acknowledged as an independent intervention unaffected by primary indication or surgery and with an often-underestimated risk for post-closure morbidity and mortality (15, 23–25). Although a vast spectrum of gastrointestinal diseases demanding an enterostomy and corresponding diverse enteric resections was included in the analysis, no negative impact of those substantial factors could be proved as relevant for the closure operation in our study. However, rectal cancer and the usual correspondingly low anterior rectum resection seem to negatively influence the post-closure outcome when waiting more than 150 days until the closure of protective loop-ileostomy. Yet, another bias must be critically considered: The closure of a protective loop-ileostomy in patients with rectal cancer is significantly more often postponed and, hence, has proportionately more cases with closure after 150 days from enterostomy.

So far, it was a silent agreement that a closure procedure should not be performed 60–90 days after installation of an enterostomy. This consensus was based on a clinical experience of patient recovery and owed to the circumstance that intra-abdominal adhesions are more manageable after about 2 months from primary surgery, and inflammation, as well as edema of the loop-ileostoma, has usually been resolved. (4, 6) Nevertheless, recent reports have even intended to curtail the time to enterostomy closure to a minimum of only a few weeks (4, 26–30). Farang et al. (29) found that early closure of loop-ileostomy within 2 weeks of index surgery of distal colorectal resection was feasible with outcomes comparable to delayed closure. Robertson et al. (30) came to the same conclusion but pointed out that further investigations are warranted with a special focus on sensitive selection strategies to identify those patients that might profit from this non-standard fast-track approach (27).

However, there are also limitations to our study that need to be considered when interpreting the results. The included number of patients (*n* = 409) is relatively small, especially when calculating the outcomes for subgroups. A retrospective analysis of clinical data, *per se*, has some limitations since the assessment of outcomes relies on others for accurate record-keeping, and because the retrospective aspect may introduce selection bias. Furthermore, the data were collected only in a single center and in a health care system with no influence of insufficient resources. This needs to be considered when our data are compared to other settings, where the closure of a protective ileostomy might be delayed due to insufficient health care resources or high costs for the patients.

In our study population, closure of a protective loop-ileostomy was feasible in 86.8% (data not shown). That means, at the same time, 13.2% of all cases failed to be reversed, and a temporarily intended enterostomy became a permanent one. Literature designates relevant risk factors that include advanced age, anastomotic leakage, metastasis, and adjuvant radiochemotherapy (4, 5, 7, 31–34). Consequently, a circumspect consideration of those predictors for non-closure, in conjunction with an overall benefit/risk analysis, is required to achieve the best outcome for each patient when deciding upon a temporary or a permanent stoma in advance of enterostomy (35, 36). Predictive tools like the nomogram, developed by Abe et al. (37), might help to identify patients with a high risk of stoma non-reversal.

## Conclusion

Protective loop-ileostomy is one of the most common interventions in abdominal surgery. Late closure (>150 days) of a protective loop-ileostomy is associated with a significantly higher rate of postoperative complications. Dysfunctional digestive problems, such as ileus, diarrhea, or stool incontinence, were the most frequent complications associated with late closure. Hence, early restitution of enteric continuity might be considered under a careful selection of patients, a thorough pre-operative assessment, and an evaluation of feasibility.

## Data Availability Statement

The raw data supporting the conclusions of this article will be made available by the authors, without undue reservation.

## Ethics Statement

The studies involving human participants were reviewed and approved by Ethical Committee of the University of Regensburg medical center under the reference number 18-104-899. Written informed consent for participation was not required for this study in accordance with the national legislation and the institutional requirements.

## Author Contributions

MH and JW: conception and design of the study. PK, ME, and SO: data acquisition. PK, ME, SO, MH, and JW: analysis and interpretation of the data. JW: drafting of the manuscript. HS, MH, and JW: revision of the manuscript. All authors had access to the study data and critically reviewed and approved the final version of the manuscript.

## Conflict of Interest

The authors declare that the research was conducted in the absence of any commercial or financial relationships that could be construed as a potential conflict of interest.

## Publisher's Note

All claims expressed in this article are solely those of the authors and do not necessarily represent those of their affiliated organizations, or those of the publisher, the editors and the reviewers. Any product that may be evaluated in this article, or claim that may be made by its manufacturer, is not guaranteed or endorsed by the publisher.
